# Development of a Multiplex Lateral Flow Immunoassay for the Detection of Antibiotics in Milk Utilizing Lyophilized Gold Nanoparticle Conjugates

**DOI:** 10.3390/bios15090592

**Published:** 2025-09-09

**Authors:** Ivan V. Maksin, Azhar Kuandykova, Darya I. Polyakova, Viktoriia A. Kesareva, Timofei A. Luzyanin, Vladislav S. Ivanov, Evgeniia I. Simonova, German A. Khunteev, Yuliya G. Kirillova

**Affiliations:** 1 Department of Biotechnology and Industrial Pharmacy, Lomonosov Institute of Fine Chemical Technologies, MIREA–Russian Technological University, 86, Vernadsky Avenue, 119571 Moscow, Russia; 2 Rapid Bio LLC, 42, bld. 1, Bolshoy Boulevard, 121205 Moscow, Russia; 3 Institute of Pharmacy and Biotechnology, Peoples’ Friendship University of Russia named after Patrice Lumumba, 8, bld. 2, Miklukho-Maklaya, 117198 Moscow, Russia; 4 Department of Veterinary Medicine, Agro–Technological Institute, Peoples’ Friendship University of Russia named after Patrice Lumumba, 8, bld. 2, Miklukho-Maklaya, 117198 Moscow, Russia

**Keywords:** immunosensor, rapid test, gold conjugate, freeze-drying, antibody, optimization, lateral flow immunoassay

## Abstract

Lateral flow immunoassays (LFAs) are widely recognized as a powerful and versatile analytical platform. Nevertheless, the development of multiplex formats remains a distinct challenge. The aim of this study was to develop a multiplex LFA using gold nanoparticles (GNPs) as a label, selected for their ease of synthesis and functionalization with biomolecules. We provide practical recommendations regarding protein–hapten synthesis, membrane selection, application buffer composition, and methods to improve the long-term stability of the freeze-dried gold conjugate. The developed assay shows good tolerance to high-fat milk, stability at elevated temperatures, and promising sensitivity, with visual detection limits of 4–100 ng/mL for β-lactams, 1–10 ng/mL for tetracyclines, 50 ng/mL for streptomycin, and 0.3 ng/mL for chloramphenicol.

## 1. Introduction

The high speed of analysis, ease of use, and cost-effectiveness make the LFA one of the most widely applied methods in resource-limited settings. Recently, interest in this technology has grown rapidly due to increasing demand from modern industries [1]. LFA has numerous applications, including the detection of hormones [2], inflammatory markers [3], cardiovascular disease markers [4], and pathogenic microorganisms [5], as well as the monitoring of drug residues in the environment [6] and food products [7].

In the food safety sector, GNP-based LFAs have been widely explored, as GNPs provide intense visible color and can be readily functionalized. Hendrickson et al. reported a GNP-based LFA for the detection of 17 fluoroquinolones (FQs) and 2 amphenicols (APs) in milk [8], while Xue et al. developed an assay for melamine (MEL), enrofloxacin (ENF), sulfamethazine (SMZ), tetracycline (TET), and aflatoxin M_1_ (AFM_1_) [9]. Peng et al. reported the detection of aminoglycosides (AMGs) across five separate test lines (TLs) with no cross-reactivity [10]. Bartosh et al. investigated how the position of test zones affects analytical performance for lincomycin (LIN), chloramphenicol (CHL), and TET [11]. Another example is a “traffic light” format using multicolor quantum dots for the detection of ofloxacin (OFL), CHL, and streptomycin (STR) in milk, which achieves 80–200 times higher sensitivity than ELISA with the same antibodies [12]. Additionally, several studies have demonstrated the application of near-infrared fluorescence (NIR)-based multiplex LFAs for milk testing. Chen et al. developed an assay capable of simultaneously detecting β-lactams, TETs, quinolones (QLs), and sulfonamides (SAs) [13], while Zhang et al. developed one for SAs, QLs, and LIN detection [14]. The use of NIR labels improves the signal-to-noise ratio by reducing interference from matrix absorption, autofluorescence, and light scattering.

Beyond milk, Chen et al. developed an LFA for the simultaneous determination of three mycotoxins in corn, rice, and peanuts [15]. In addition to line-based formats, Adunphatcharaphon et al. developed a multiplex fluorescent microarray LFA for the simultaneous detection of five mycotoxins in rice, employing dot-based test zones instead of conventional lines [16]. Since dot spots occupy considerably less space than test lines, they may allow for higher multiplexing capacity per strip. However, increasing the number of test zones significantly complicates the visual interpretation of results and necessitates the use of a reader device for accurate analysis. To address this limitation and enable instrument-free result interpretation, color-coded labels can be employed, such as dyed cellulose [17] or latex nanoparticles [18], as well as plasmon-controlled nanocomposites (PINs) [19].

The demand for corresponding diagnostic assays comes mainly from farms and processing enterprises for the quality control of raw materials, finished products, and semi-finished products. The analysis should ensure high sensitivity, rapid results, and ease of interpretation and use [20]. For small molecule detection and quantification, instrumental analysis is considered the gold standard due to its high sensitivity [21]. However, the application of this method is often constrained by high operational expenses, the need for skilled personnel, and complicated sample preparation. In contrast, LFAs provide a rapid and user-friendly alternative for on-site screening, albeit typically with lower sensitivity and specificity.

Several comprehensive studies have addressed general design principles and key challenges in LFA development [22,23], particularly in relation to the improvement of analytical sensitivity [24,25]. In particular, for the detection of antibiotics, haptens play a crucial role. Haptens, small molecules that become immunogenic only when conjugated to carrier proteins, are widely used for the detection of low-molecular-weight compounds. Rational hapten design, which tailors molecular structure to mimic the target, is critical for producing antibodies with high specificity and affinity [26] and has a direct impact on biosensor sensitivity and specificity [27,28].

This study aimed to develop a multiplex LFA for the simultaneous detection of β-lactams, TETs, STR, and CHL using GNPs instead of fluorescent markers as labels, so that the visual signal can be easily interpreted with the naked eye without specialized equipment. Until 2023, these were the only antibiotic groups subject to mandatory screening in raw milk under TR CU 033/2013 (Technical Regulations of the Customs Union “On the safety of milk and dairy products”). This technical regulation establishes mandatory safety requirements for milk and dairy products put into circulation in the customs territory of the Customs Union (Republic of Belarus, the Republic of Kazakhstan, and the Russian Federation), and they remain among the most monitored residues due to their regulatory significance and widespread use in dairy farming. Various assay parameters need to be optimized, including, but not limited to, GNP size [15], bioreceptor conjugation method [29], protein–hapten conjugate composition [27,28], membrane material [22], and excipient formulation [30], as they can significantly impact assay performance and shelf life. The optimization process was based on known scientific and technological principles, emphasizing the identification and resolution of “bottleneck” issues not thoroughly described in the literature. The selected parameters are guided by scientific rationale and considerations of scalability and commercial availability.

## 2. Materials and Methods

### 2.1. Materials

Sodium borohydride, sodium hydride (60% dispersion in mineral oil), N-Boc-ethanolamine, triisopropylsilane (Acros Organics, Geel, Belgium). Tris-hydroxypropyltriazolylmethylamine (THPTA) (Lumiprobe, Moscow, Russia). A 50% water solution of glutaraldehyde (GA), 1-Ethyl-3-(3-dimethylaminopropyl)carbodiimide (EDC), dextran 40000, N-hydroxysuccinimide (NHS), 4-(2-hydroxyethyl)-1-piperazineethanesulfonic acid (HEPES), Brij-35, Tween-80, Pluronic F-127, 3-((3-cholamidopropyl) dimethylammonio)-1-propanesulfonate (CHAPS), cloxacillin sodium salt, amoxicillin, ceftiofur sodium salt, oxytetracycline, tetracycline hydrochloride for conjugation (Molekula, London, UK). 2-(N-morpholino)ethanesulfonic acid (MES), a 37% solution of formaldehyde, sucrose, trehalose dihydrate, ampicillin sodium salt (AMP, Neofroxx, Einhausen, Germany). Hydroxylamine hydrochloride, NP-40, streptomycin for conjugation (Panreac, Barcelona, Spain). Propargyloxypropionic acid hydrazide (Primetech, Minsk, Belarus). HAuCl_4_ (Aurat, Moscow, Russia). Propargyl bromide, 2-iminothiolane, 1-hydroxybenzotriazole, 4-dimethylaminopyridine (TCI, Tokyo, Japan). Chloramphenicol succinate (Santa Cruz Biotechnology, Dallas, TX, USA). Sulfosuccinimidyl 4-(N-maleimidomethyl)cyclohexane-1-carboxylate (Sulfo-SMCC, Thermo Fisher Scientific, Waltham, MA, USA). Chlortetracycline (Thermo Fisher Scientific, Waltham, MA, USA), CHL, penicillin sodium salt, doxycycline hyclate, tetracycline hydrochloride, streptomycin sulphate for sensitivity and specificity evaluation, (FGBU “VGNKI”, Moscow, Russia). All other reagents were obtained from domestic manufacturers and were of at least chemically pure grade. Goat anti-mouse immunoglobulins (GAMI), goat anti-chicken immunoglobulins (GACHI), chicken IgY, mouse IgG (Arista biologicals, Allentown, PA, USA). Fatty-acid-free bovine serum albumin (BSA, Proliant, Feilding, New Zealand). Mouse monoclonal antibodies against tetracycline, chloramphenicol, streptomycin, histidine tag, as well as recombinant histidine-tagged penicillin-binding protein (PBP) were received from Eastmab (Nanjing, China). Backing card SM31-40 and absorbent pad CH37 (Kinbio, Shanghai, China) were used in all experiments. Tested sample pads and nitrocellulose membranes were purchased directly from the manufacturers. Microwells (Sovtech, Berdsk, Russia). Milk samples were purchased from a Moscow retail network.

### 2.2. The Synthesis of GNPs

GNPs were synthesized according to the method described by Kumar et al. [31]. Initially, a seed solution was prepared via citrate reduction of chloroauric acid. The resulting nanoparticles were then diluted with deionized water at ratios of 1:2, 1:4, 1:10, and 1:25. To each diluted solution, 0.5 mL of a 1% sodium citrate solution, 0.1 mL of a 5% chloroauric acid solution, and 0.25 mL of a 0.5% hydroquinone solution were added sequentially with stirring at 700 rpm. The reaction mixture was stirred continuously for 4 h, after which the resulting GNP solution was used for further applications. Spectra of the GNP solutions were recorded using a UV5Nano instrument (Mettler Toledo, Greifensee, Switzerland).

### 2.3. The Conjugation of GAMI and GACHI with GNPs by Passive Adsorption

The optimal pH level was determined by following the published protocol [32]. A volume of 1 mL of 2 μg/ml GAMI solution was added to 10 mL of a GNP solution, adjusted to a pH of 8. The mixture was then placed in an orbital stirrer and allowed to incubate for a period of 30 min. Subsequently, 0.4 mL of a 10% BSA solution was added to the mixture, which was then incubated for an additional 30 min. The mixture was centrifuged for 30 min at 5000× *g*. The supernatant was collected, and the sediment was re-dissolved in 1 mL of 4 mM Tris buffer solution with a pH of 8, containing 5% sucrose and 0.1% BSA (unless otherwise specified). The optical density (OD) of the conjugate solution was measured, and if the OD exceeded 10, the solution was diluted accordingly to achieve an OD of 10. Therefore, conjugates of GNPs with GAMI (GNP-GAMI) were obtained. The same protocol was used to prepare GNP-GACHI conjugates.

### 2.4. Conjugation of GNP-GAMI with Antibiotic-Specific Receptors

A specific amount of the respective antibody (anti-TET, anti-STR, anti-histidine or anti-CHL) was added to the GNP-GAMI solution and thoroughly mixed for 2 min. The optimal antibody concentration for each conjugate was determined during the optimization process. Subsequently, a solution of mouse IgG was added to achieve a final concentration of 20 μg/mL in the mixture. Consequently, GNP conjugates with the respective antibodies were prepared and labeled as GNP-anti-TET, GNP-anti-STR, GNP-anti-His, and GNP-anti-CHL.

A specific amount of PBP was added to the GNP-anti-His solution and thoroughly mixed for 2 min. The resulting conjugate was labeled as GNP-anti-Blac.

### 2.5. Protein–Hapten Synthesis and Analysis

Detailed procedures for the synthesis of protein–haptens are described in the Supplementary Materials.

NMR spectra of hapten **5** and its intermediate **3** (^1^H, 300 MHz; ^1^3C, 75 MHz) were acquired on a Bruker DPX-300 spectrometer at the Shared Science and Training Center for Collective Use, RTU MIREA (Figures S1–S3).

A mass spectrometric analysis of protein–hapten conjugates was conducted in positive ion detection mode on a RapifleX instrument (Bruker, Bremen, Germany) at the Advanced Mass Spectrometry Core Facility of the Skolkovo Institute of Science and Technology (Figures S4–S6). Spectra were visualized using Origin 9.8 software (OriginLab, Northampton, MA, USA).

### 2.6. Preparation of the Test Strips

A Biodot ZX1010 dispenser (BioDot, Irvine, CA, USA) was utilized to apply conjugates comprising conjugates of BSA with AMP, STR, CHL, and TET to the designated test zones of nitrocellulose membranes, with chicken IgY being dispensed to the control zone. The membranes with applied reagents were dried in an incubator at 45 °C for 1 h. Thereafter, the membranes were stored under conditions of low humidity.

### 2.7. Lyophilization of GNP Conjugates

GNP-anti-TET, GNP-anti-STR, GNP-anti-Blac, GNP-anti-CHL, and GNP-GACHI were mixed and dispensed into the 8-well strips, then frozen and lyophilized for 40 h. The strips containing the lyophilized product were sealed with caps, placed into zip-lock bags with silica gel, and stored at 4 °C.

### 2.8. Assay Procedure

**Figure 1:**
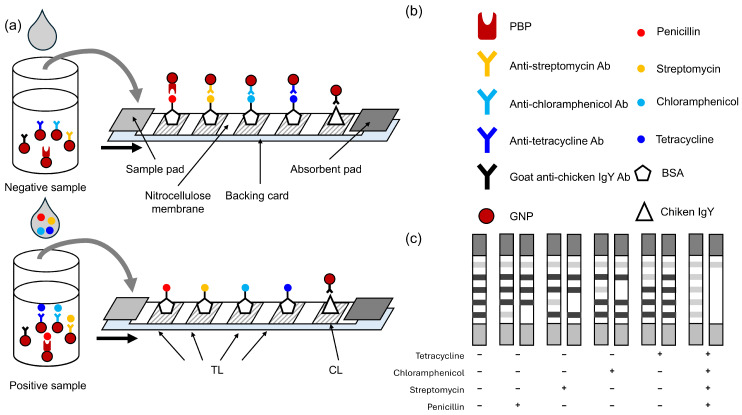
Schematic diagram of the developed competitive LFA for detecting antibiotics. (**a**) Negative sample; (**b**) positive sample; (**c**) the assay result interpreting scheme.

The immunochromatographic test strip was designed as shown in Figure 1. The application of BSA-ampicilline, BSA-streptomycin, BSA-chloramphenicol, BSA-tetracycline, and chicken IgY to the nitrocellulose resulted in the formation of four TLs and a control line (CL), respectively. To the lyophilized GNP conjugates, 200 µL of an untreated (undiluted and without additives) milk sample was added. The well was then incubated for 5 min at 40 °C in a thermo-incubator (Allsheng, Hangzhou, China). Following the incubation period, the test strip was immersed into the well for a duration of 5 min. After incubation, the test strip was placed in the well for 5 min, allowing the mixture to migrate along the strip by capillary action. Subsequently, the test strip was removed from the solution and the sample pad membrane was detached to stop the liquid flow. In the absence of analytes in the sample (Figure 1a), receptors bind to antibiotics bound to BSA. This binding leads to the accumulation of GNPs on the TL and the subsequent appearance of coloration. Analytes present in the sample inhibit the GNP conjugate binding to the protein–hapten conjugate on the TL, causing weak or absent TL coloration (Figure 1b). The test result is determined by comparing the intensity of the TL and CL (Figure 1c). In cases where the TL is more intense than the CL, the result is negative. In cases where the TL is found to be less intense than the CL, or where these two variables are found to have comparable intensity levels, the result is deemed to be positive. The visual limit of detection (vLOD) was defined as the lowest analyte concentration at which the intensity of the TL was comparable to or lower than that of the CL, as determined by visual inspection.

Since assay conditions and milk sample composition can affect the visual interpretation of results, we deemed it necessary to use the CL as a reference for comparing coloration intensity. In conventional designs, the control line is formed by antibodies specific to the analytical antibodies, which allows monitoring of their binding [22]. However, in our multiplex format this approach would cause variation in CL coloration depending on how much conjugate is retained on the TL. Therefore, as a practical solution at the prototyping stage, we introduced an additional reagent (GNP–GACHI) that binds to chicken IgY, ensuring stable CL coloration independent of the analytical conjugate signal.

The coloration intensity of the test strip lines was measured using a TSR-100 test-strip reader (Allsheng, Hangzhou, China). Calibration curves were constructed based on the obtained line intensity values using the Origin 9.8 software package. Calibration curves were fitted using a four-parameter logistic (4PL) regression model. The instrumental limit of detection (iLOD) was defined as three times the standard deviation (3SD) of the negative control signal, while the limit of quantification (LOQ) was defined as 10SD. Both parameters were calculated with respect to the fitted calibration curve.

## 3. Results and Discussion

### 3.1. Synthesis of GNPs

For the synthesis of GNPs, we employed a two-step method [31] that involves the preparation of seed nanoparticles—small-sized particles obtained using the Frens method by reducing chloroauric acid with sodium citrate—followed by gold condensation on the pre-synthesized nuclei. The two-step seed-mediated approach enables the production of monodisperse nanoparticles at room temperature with precise control over their size. The size of the resulting nanoparticles was controlled by adjusting the amount of seeds in the solution. The nanoparticle sizes were determined by correlating the wavelength of maximum absorbance (Figure S7a) with data from the literature [33]. Accordingly, particle sizes of approximately 25, 45, 50, 60, and 80 nm were obtained.

### 3.2. Conjugation of GNP with Bioreceptors

For the detection of β-lactams, we chose recombinant PBP because of its broad specificity and ability to recognize only molecules with an intact β-lactam ring [34]. For the detection of STR, TET, and CHL, we chose monoclonal antibodies due to better lot-to-lot reproducibility compared to polyclonal antibodies. Several recent literature reviews have covered the importance of bioreceptor orientation in terms of sensitivity in immunoassays [35,36], and in LFA in particular [29]. For antibody immobilization, we employed anti-mouse antibodies specific to the Fc region. This allows the Fab region of the antibody to be oriented towards the solution, reducing steric hindrance. A detailed description of anti-mouse-assisted immobilization will be covered in a forthcoming publication [37].

Recently, we demonstrated a 16-fold sensitivity enhancement of LFA for penicillin by utilizing anti-histidine-tag-assisted immobilization of recombinant His-tagged PBP on gold nanoparticles compared to its non-oriented immobilization using the common physical adsorption approach [32]. For PBP immobilization, in this study, we employed a double-linker system, where anti-his-tag antibodies were first captured on the gold nanoparticle surface with anti-mouse antibodies, and then his-tag-labeled PBP was added. Such a “skyscraper” approach reduces the amount of anti-his-tag antibodies needed due to better antibody orientation.

The selection of GNP size was based on the sensitivity observed with conjugates of GNP-anti-STR. Numerous studies show that using large GNPs can improve assay sensitivity [38,39,40]. From a manufacturing technology perspective, using larger GNPs can be more advantageous, as it allows the use of less powerful centrifuges and reduces mechanical wear on the equipment. However, larger nanoparticles tend to be less stable during storage. As shown in Figure S7b, all tested nanoparticle sizes provided the required sensitivity. At an STR concentration of 200 ng/mL, corresponding to its maximum residue limit (MRL) in milk, the TL completely disappears. Notably, at a lower concentration of 25 ng/mL, the reduction in coloration intensity is more pronounced with the use of 50 nm GNPs. For subsequent experiments, 50 nm GNPs were selected as a compromise between efficient particle sedimentation during conjugate preparation and nanoparticle stability in solution.

### 3.3. Comparison of Protein–Hapten Synthesis Protocols

Since antibiotics cannot be directly immobilized on nitrocellulose membranes, they must first be conjugated to a carrier protein. The conjugation method largely depends on the chemical nature of the hapten and is limited by the reactive groups available for coupling under aqueous conditions.

**Figure 2:**
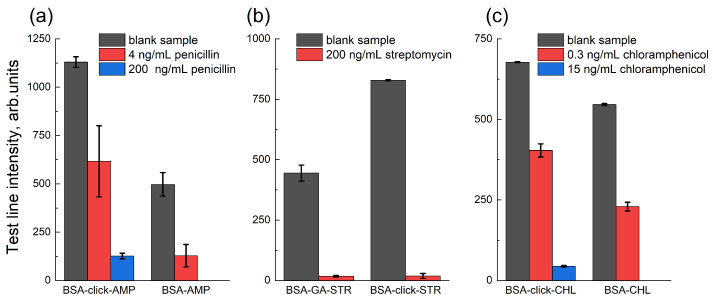
Comparison of (**a**) BSA-ampicillin, (**b**) BSA-streptomycin, and (**c**) BSA-chloramphenicol coupling strategies. Values shown are means of three runs (n=3).

Penicillin antibiotics share a conserved structural core consisting of a β-lactam ring fused to a thiazolidine ring, which carries a carboxyl group at the 3-position. The β-lactam moiety is linked via an amide bond to a variable side chain. A straightforward one-step synthesis of a conjugate with any penicillin antibiotic can be achieved by coupling the carboxyl group to protein amino groups using an activated ester method. We performed direct conjugation of AMP to BSA using carbodiimide chemistry in the presence of EDC and NHS, yielding the BSA-AMP conjugate. In such a conjugate, the carrier protein may shield the conserved penicilloic acid residue and, consequently, the β-lactam ring, which is the key recognition site for penicillin-binding protein. As an alternative, we employed a two-step conjugation strategy that avoids this drawback. AMP was first coupled with the sulfo-SMCC linker via its side-chain amino group and then coupled by Michael addition to BSA pre-functionalized with thiol groups. The resulting BSA-click-AMP conjugate produced a significantly higher signal output (Figure 2a) than the BSA-AMP conjugate.

STR is an AMG containing guanidine groups, a secondary amine, and an aldehyde group. STR was coupled to BSA using GA as a bifunctional linker, producing the BSA-GA-STR conjugate. The coupling of STR with GA can occur via both the amine and guanidine residues. Due to the presence of multiple reactive groups in both the antibiotic and the carrier protein, this approach may result in heterogeneous conjugation and the formation of cross-linked products. Furthermore, reduction of the intermediate Schiff base with sodium borohydride may also reduce functional groups on streptomycin, potentially impairing hapten recognition by the bioreceptor.

As a more selective approach for STR conjugation, we employed copper-catalyzed azide-alkyne cycloaddition (CuAAC) click chemistry [41]. STR was selectively modified through its aldehyde group with the hydrazide of propargyloxypropionic acid, followed by a click reaction with BSA-azide, yielding the BSA-click-STR conjugate. The BSA-click-STR conjugate exhibited a twofold higher signal output compared to BSA-GA-STR (Figure 2b). The homogeneous nature of BSA-click-STR, with defined conjugation through specific functional groups, makes it the preferred choice for assay development.

CHL lacks reactive groups suitable for conjugation under aqueous conditions. Therefore, the preparation of CHL–protein conjugates requires the use of its derivatives. A convenient derivative is commercially available CHL succinate, which can be conjugated to proteins via EDC/NHS activation. Additionally, we synthesized an alkyne-containing hapten **5** for conjugation using CuAAC. However, there was no significant difference in signal intensity between BSA-CHL and BSA-click-CHL (Figure 2c). It is likely that the most characteristic nitrophenyl and dichloroacetamide groups of chloramphenicol are similarly accessible for recognition in both conjugates. The slight increase in signal observed with BSA-click-CHL may be attributed to improved biospecific recognition due to the longer spacer arm. Nevertheless, the observed positive effect does not warrant the use of the more complex three-step synthesis of hapten **5** (Figure S8).

The BSA-TET conjugate was synthesized via the Mannich reaction by treating a mixture of tetracycline hydrochloride and BSA with formaldehyde. Alternatively, a two-step conjugation was performed using tetramethylbenzidine as a homobifunctional linker, involving the formation of a diazonium salt [42]. Unfortunately, the resulting BSA-TMB-TET conjugate exhibited pronounced purple coloration, which interfered with the visual interpretation of the assay.

**Figure 3:**
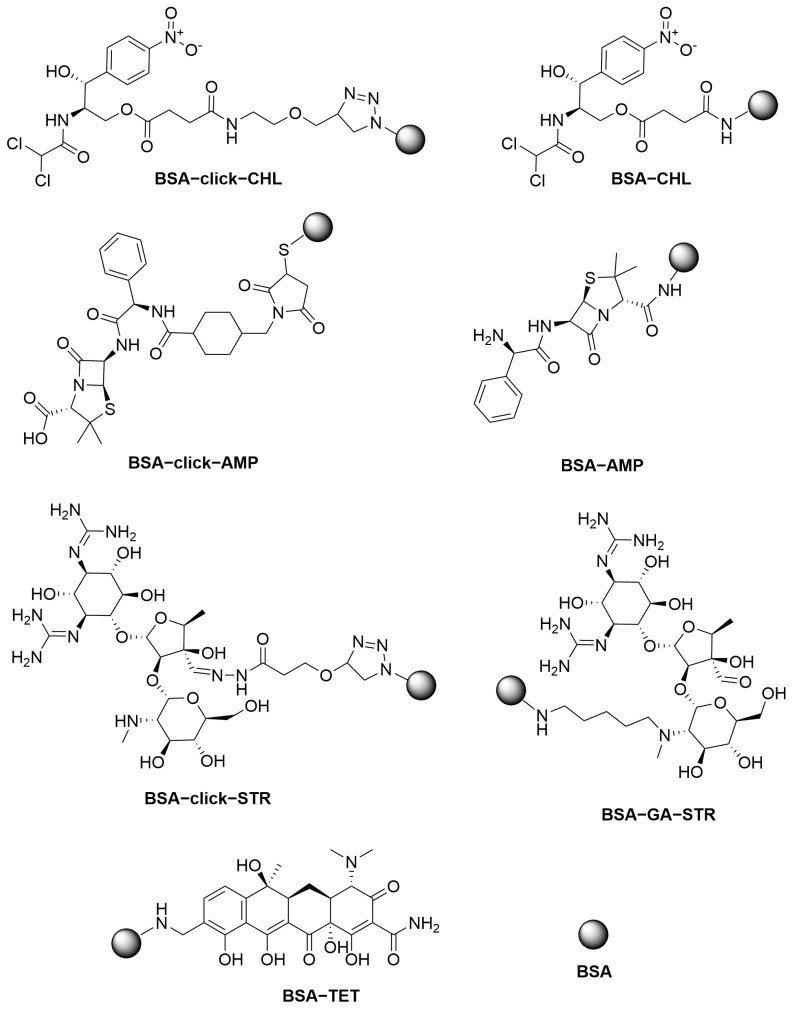
Chemical structures of the synthesized protein–hapten conjugates.

Therefore, for the optimization stage, the BSA-click-AMP, BSA-click-STR, BSA-CHL, and BSA-TET conjugates were selected. The structures of the resulting conjugates are illustrated in Figure 3.

### 3.4. Buffer Composition for Protein–Hapten Application

The composition of the buffer used for applying reagents onto the nitrocellulose membrane was optimized during the development of the four TLs. Several issues were encountered. The primary problem was uneven liquid flow through TLs during analysis, with occasional complete stoppage of flow. We hypothesize that this issue was caused by the hydrophobic nature of the protein–antibiotic conjugates, with the most pronounced effect observed for the BSA–tetracycline line. To address this, it was necessary to make the TL zones more hydrophilic.

**Figure 4:**
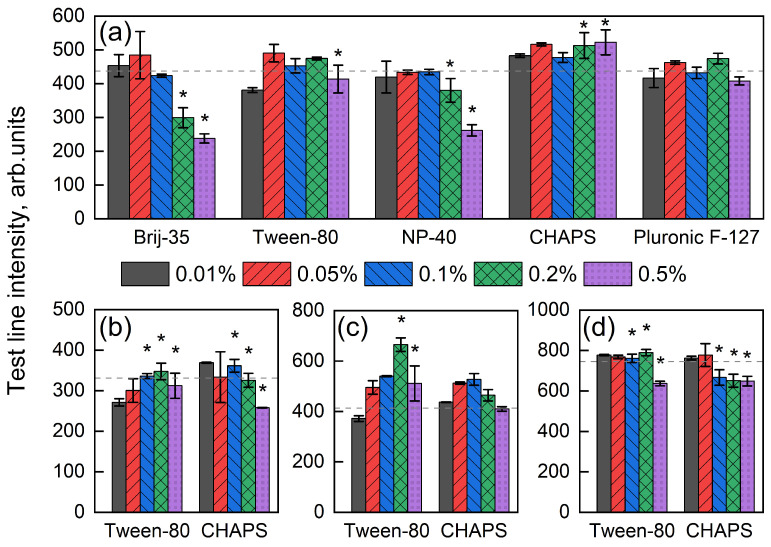
Effect of detergent concentration on line coloration intensity and liquid flow through the lines: (**a**) BSA-TET, (**b**) BSA-click-AMP, (**c**) BSA-CHL, (**d**) BSA-click-STR. Asterisks (*) indicate detergent concentrations at which no flow hindrance was observed. The horizontal dashed line represents the coloration intensity of the lines without detergent addition. Values shown are the means of three runs (n=3).

We hypothesized that adding amphiphilic molecules such as detergents can address the problem (Figure 4). It is believed that the strong affinity of proteins to nitrocellulose arises from dipole interactions between the nitro groups of the polymer and the carbonyl groups in peptide bonds, as well as interactions with hydrophobic amino acid residues of proteins [43]. However, excessive detergent levels may disrupt conjugate–nitrocellulose interactions, leading to conjugate desorption and impaired signal response. The concentration effect of several detergents commonly used in immunoassays was studied for BSA-TET performance evaluation: the nonionic detergents Brij-35, Tween-80, NP-40, and Pluronic F-127, and the ionic detergent CHAPS (Figure 4a).

Brij-35 and NP-40 facilitated flow only at concentrations that caused a significant decrease in line coloration intensity. Pluronic F-127 had no noticeable effect on either coloration intensity or flow quality. In contrast, Tween-80 and CHAPS improved flow without substantially reducing coloration intensity, with CHAPS even slightly enhancing it.

To evaluate the concentration effect of detergents on the other lines, Tween-80 and CHAPS were selected. The results differed from those observed for BSA-TET, generally requiring lower detergent concentrations. Notably, for BSA-CHL, the addition of 0.2% Tween-80 improved both flow and coloration intensity by approximately 1.5 times (Figure 4c).

We hypothesize that the optimal choice of detergent and its concentration depends on the hydrophobicity of the conjugate, which is largely determined by the properties of the hapten, the BSA-to-hapten ratio, and the overall concentration. For subsequent experiments, the optimal detergents were considered to be 0.2% Tween-80 for BSA-CHL, BSA-click-STR, and BSA-AMP, and 0.2% CHAPS for BSA-TET. The time required for the sample to migrate to the end of the strip decreased from 140 s to 110 s (Figure S9), and the liquid front was distributed evenly.

#### 3.4.1. Selection of Nitrocellulose Membrane

The choice of nitrocellulose membrane depends on sample type, nanoparticle size, and required sensitivity. Researchers often use pore size and wicking time (the time for water to travel 4 cm) as selection criteria. Wicking time (s/4 cm) is inversely related to flow rate. Slower membranes can increase sensitivity by allowing more interaction time between reagents and analytes. However, since this assay uses a pre-incubation step, slow membranes offer no advantage. Fast-flow membranes are preferable for viscous samples like milk, as they reduce flow hindrance and shorten assay time.

**Figure 5:**
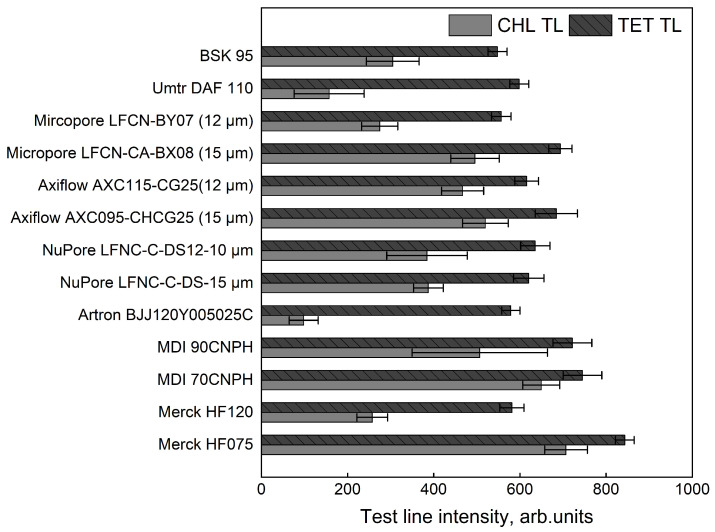
Comparison of test line coloration intensity for BSA-CHL and BSA-TET conjugates applied to different nitrocellulose membranes. Values shown are the means of three runs (n=3).

To identify the most suitable membrane, we compared 15 fast-flow membrane types by TL coloration intensity (Figure 5). BSA-TET and BSA-CHL were used for coating, as these lines showed the most pronounced flow hindrance. The selected detergent (see Section 3.4) was added to the dispensing solution. For BSA-CHL, coloration intensity varied greatly between membranes, while for BSA-TET, differences were less significant. These results show that optimal buffer compositions are not universal for all nitrocellulose membranes, so membrane selection is largely empirical. Faster membranes generally produced more intense lines, likely due to reduced interference from milk fat. MDI 70CNPH and Merck HF075 gave the highest signals. MDI 70CNPH was chosen for further experiments because of its lower cost.

#### 3.4.2. Matrix Effect Tolerance

When testing milk samples with high fat contents (3.4–6%), we observed a marked reduction in the CL intensity, while the TL remained largely unaffected. Since LFA result interpretation relies on comparison of the intensity between CL and TLs, reduced CL intensity may lead to inaccurate results. Therefore, it is important to maintain consistent coloration of both the CL and TL across milk samples with varying fat contents.

To address this matrix effect, sample dilution is commonly used before analysis [7]. However, dilution lowers analyte concentration and reduces assay sensitivity. Our goal was to improve the CL coloration in high-fat milk samples without additional dilution.

We explored the use of different sample pad materials. As suggested by Ahlstrom [44], optimizing pad composition can enhance fluid flow and reduce fat interference. We tested various commercially available pads (Table 1). Most performed similarly, with only minor differences in flow, but some reduced TL intensity, likely due to pretreatment chemicals. Good adhesion to the backing card was also important, as most pads detached easily during handling. None of the tested materials tolerated high-fat samples well—the CL intensity still dropped sharply (Figure S10). We selected the MDI FR2(0.8) membrane for further experiments due to its handling and acceptable performance.

We hypothesized that adding a detergent to the sample could reduce the masking effect of fat. A concentrated aqueous solution of Triton X-100 was added to the gold conjugate solution before lyophilization. As shown in Figure S11, Triton X-100 significantly improved the CL intensity, making it comparable to that in low-fat milk. However, a decrease in TL intensity (except for the TET line) was observed in low-fat milk. If no additional detergents were added to the protein–hapten conjugates, adding Triton X-100 to the GNP conjugate did not reduce TL intensity in low-fat milk, while still improving the CL intensity in high-fat milk.

We speculate that in high-fat samples, the detergent solubilizes fat, reducing viscosity and improving reagent flow at the CL. In low-fat milk, excess detergent may partially solubilize and desorb protein–hapten conjugates from the membrane (especially since other detergents were used during the application), resulting in decreased TL intensity.

Based on these results, we used gold conjugates with Triton X-100 and omitted detergents from the deposition buffer for the BSA-click-AMP, BSA-CHL, and BSA-click-STR conjugates in further experiments. In Table 2, we summarized our observations regarding the utilization of detergents.

### 3.5. Optimization of Immunoreagent Concentrations

The next step was to optimize the concentrations of immunoreagents to achieve the required sensitivity while maintaining clear visual detection of the TLs. Optimization was performed for both the coating concentration of protein–hapten conjugates (Figure S12) and the concentration of specific bioreceptors used for GNP conjugate preparation (Figure S13). The process was based on titration experiments. Selected optimal concentrations are marked with a check marks on the figures.

The following considerations guided the optimization:

- The intensity of the TLs should be significantly higher than that of the CL in negative samples (TL/CL intensity ratio score > 2) and lower in positive samples (TL/CL intensity ratio < 1) to ensure accurate visual interpretation of the results.
- The minimum concentration of reagents should be used that still provides a strong signal, thus minimizing the consumption of costly reagents.

### 3.6. Optimization of the Lyophilization Excipients

There are two main approaches for introducing a receptor–label conjugate into an assay. The first involves dispensing the conjugate onto a conjugate pad, followed by drying and incorporation into the test strip. In this approach, the conjugate re-dissolves immediately upon immersion of the strip into the sample, thereby simplifying the assay procedure. The second approach involves placing the receptor–label conjugate separately, either in liquid or lyophilized form. In this case, the assay begins with mixing the conjugate and the sample or extract, followed by incubation. After incubation, the test strip is immersed into the mixture. This format provides improved kinetic conditions for the formation of the receptor–analyte complex, as the reaction is no longer limited by the diffusion constraints of the porous structure of the test strip membrane [7]. As a result, the second approach generally offers better sensitivity.

The pre-incubation approach requires the receptor–label conjugate to be either stable in liquid form for extended periods or formulated as a freeze-dried product. It is well established that biomolecules are generally better preserved in a dry form, provided the formulation is properly optimized. To the best of our knowledge, none of the available publications provides a detailed description or justification for the choice of excipients used in the formulation of freeze-dried GNP–receptor conjugates.

Lyophilization is typically divided into three stages: (i) freezing below the eutectic or glass transition point to preserve the structure, (ii) primary drying (removal of the bulk of the water), and (iii) secondary drying (desorption of bound water). The formulation of the solution includes several components collectively referred to as excipients [45]. These excipients typically include bulking agents, which facilitate the formation of an elegant lyophilized “cake”; stabilizers, which protect proteins from denaturation and aggregation during freezing, drying, and storage; and surfactants, which help stabilize proteins at the ice–air and/or ice–solution interfaces. Buffering agents are also added to maintain a stable pH throughout the process.

Sucrose and trehalose are among the most commonly used amorphous cryo- and lyoprotectants. However, both have relatively low glass transition temperatures (*T’_g_* is about minus 30 °C). *T’_g_* is usually several degrees lower than the collapse temperature (*T_c_*). If the primary drying temperature exceeds *T_c_*, the lyophilizate may lose its elegant, porous “cake” structure, which inhibits subsequent sublimation. To increase *T’_g_* and improve stability during storage, sucrose and trehalose are often combined with excipients possessing higher *T’_g_*, such as dextran or cyclodextrins. Mannitol is frequently added to improve the physical appearance and structure of the lyophilizate [46].

Thus, the primary focus of this part of the study was to determine the optimal concentration of stabilizers. For comparison, we selected the most widely used carbohydrates: sucrose, trehalose, dextran, 2-hydroxypropyl-β-cyclodextrin, and mannitol. Tris buffer was chosen as the buffering system. Based on these components, 12 formulations were prepared and used as lyophilization buffers for GNP–receptor conjugates (Table 3).

To evaluate the stabilizing effect, an accelerated aging test (AAT) was employed—storage at a temperature exceeding the intended storage temperature. This approach to predicting the shelf life of a product is grounded in the Arrhenius equation and is therefore known as an “Arrhenius accelerated stability study” [47]. The prepared lyophilizates were stored at 37 °C, and the functionality of the conjugates was assessed at various time intervals. To select the optimal formulation for the stability of all gold conjugates, we used the Harrington desirability function, which is well-suited for multiparameter optimization [48].

**Figure 6:**
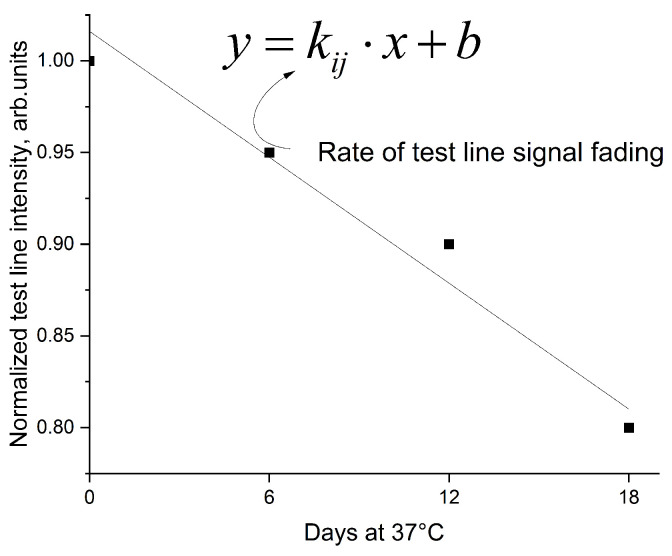
Reduction in the TL coloration intensity of the lyophilized conjugate during the AAT.

For each gold conjugate (GNP-GACHI, GNP-anti-TET, GNP-anti-STR, GNP-anti-CHL, and GNP-anti-Blac), the stability was characterized by the slope coefficient $k_{ij}$ of the linear regression describing the change in TL intensity over time for the *j*-th buffer formulation (Figure 6). A less negative (closer to zero) slope indicates better stability (i.e., less signal loss during storage).

The individual desirability values $d_{ij}$ were calculated using Equation (1):

(1) $$d_{ij}=\left\{\begin{array}{cc} 0, & k_{ij}\leq k_{min\;i}\\ \frac{k_{ij}-k_{min\;i}}{k_{max\;i}-k_{min\;i}}, & k_{min\;i}<k_{ij}<k_{max\;i}\\ 1, & k_{ij}\geq k_{max\;i} \end{array}\right.$$

where $k_{min\;i}$ and $k_{max\;i}$ are the minimum and maximum slope values observed for the *i*-th conjugate across all tested formulations. Thus, $d_{ij}$ reflects how effectively a particular buffer formulation preserves the stability of each conjugate, with 1 indicating the best result and 0 the worst.

The overall desirability $D_{j}$ was calculated using Equation (2).

(2) $$D_{j}={\left(d_{1j}\times d_{2j}\times \ldots \times d_{nj}\right)}^{1/n}$$

Composition Nº3 demonstrated the highest cumulative stability; however, in terms of absolute values, compositions Nº6 and Nº9 were only slightly inferior (Table 3). Given that cyclodextrin and dextran are more expensive than sucrose, composition Nº6 was selected for further work.

### 3.7. Specificity and Sensitivity Evaluation of the Assay

**Figure 7:**
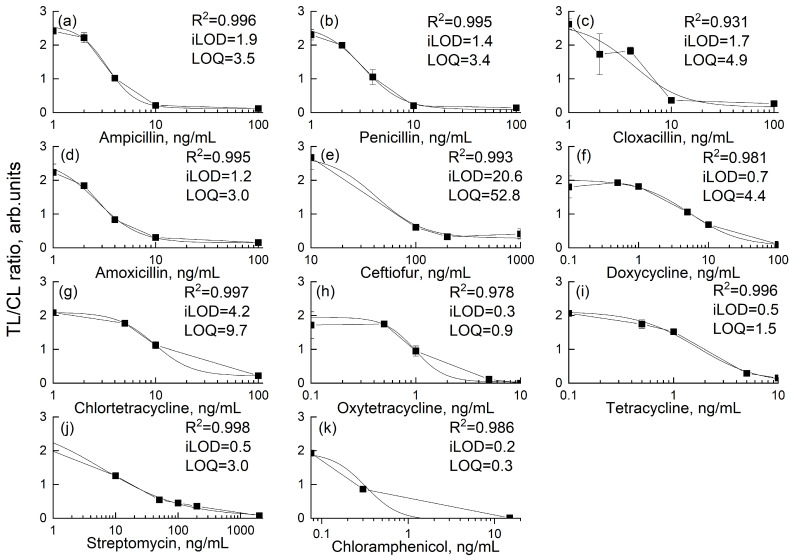
Calibration curves of control-to-test line intensity ratio vs. antibiotic concentration in milk for (**a**) ampicillin, (**b**) penicillin G, (**c**) cloxacillin, (**d**) amoxicillin, (**e**) ceftiofur, (**f**) doxycycline, (**g**) chlortetracycline, (**h**) oxytetracycline, (**i**) tetracycline, (**j**) streptomycin, and (**k**) chloramphenicol. Values shown are the means of three runs (n=3).

Upon the addition of antibiotics, even at concentrations 50 times higher than the established MRL, the TLs corresponding to the respective antibiotics showed a complete absence of coloration. At the same time, no noticeable changes in the intensity of the remaining TLs were observed, indicating the high specificity of the assay (Figure S14). The LOD was determined by visually inspecting the test strip. It was defined as the lowest concentration of analyte at which the TL intensity was equal to the CL. LOQ and iLOD were calculated based on the calibration curves (Figure 7). The method exhibits promising sensitivity, indicating its potential as a prototype for meeting TR CU, EU, and China MRLs (Table 4).

## 4. Conclusions

The multiplex lateral flow immunoassay for the simultaneous detection of β-lactams, streptomycin, tetracyclines, and chloramphenicol was developed as a result of assay optimization. The final assay is reliable, visually interpretable, and suitable for on-site screening of antibiotic residues in milk. Its sensitivity and specificity meet regulatory requirements, supporting its practical application in food safety monitoring and quality control.

A significant part of the study focused on the synthesis of suitable protein–hapten conjugates. To achieve high assay sensitivity, we determined that (i) the functional groups responsible for specific interaction with the bioreceptor should remain exposed and unaltered; (ii) preference should be given to the use of selective click reactions, such as CuAAC and maleimide–thiol bioconjugation; and (iii) the conjugation should introduce a spacer arm to facilitate effective bioreceptor recognition.

Matrix tolerance was another important aspect. We found that the hydrophobicity of protein–hapten conjugates can hinder sample flow through the test strip. The incorporation of detergents improved flow quality. However, excessive detergent caused partial desorption of the conjugate from the membrane. Ultimately, detergent was included only in the freeze-dried conjugate formulation, though adding detergent to TL reagents may be beneficial in other contexts.

The choice of nitrocellulose membrane and sample pad material also influenced assay performance. Due to the unique properties of protein–hapten conjugates and proprietary membrane treatments, precise recommendations regarding surfactant type and concentration cannot be universally provided.

Stabilization of gold nanoparticle–receptor conjugates by lyophilization was also addressed. The Harrington desirability function proved to be a useful tool for optimizing excipient combinations in a multicomponent system, enabling improved stability, as demonstrated in accelerated aging tests. It should be noted that the application of the desirability function is not limited to stability testing but can also be effectively employed in other optimization tasks, such as improving conjugation efficiency, enhancing signal intensity, or balancing multiple performance parameters in complex assay development.

During assay development and optimization, we recommend the use of a test strip reader. This device provides objective, quantitative results and greatly facilitates the optimization of reagent concentrations and assay conditions compared to subjective visual assessment or time-consuming test strip scanning and image analysis in software. Importantly, the final assay is designed for visual interpretation and does not require a reader for routine use.

The approaches and optimization strategies described here can serve as a practical guide for researchers developing multiplex lateral flow immunoassays, especially for complex matrices such as milk. By sharing detailed insights on reagent preparation, membrane selection, buffer formulation, and conjugate stabilization, we aim to facilitate the development of robust and sensitive rapid tests for food safety and related applications.

## Figures and Tables

**Table 1:** Overview of sample pad materials and their performance characteristics.

Sample Pad Material	Performance Characteristics
Kinbio BT50	Weak adhesive to backing card
Kinbio BT53	Weak adhesive to backing card
Kinbio BT40	Fast flow, strong adhesive to backing card
Axiflow PSP35	CHL line shows weak coloration
Axiflow PSP35+	CHL line shows weak coloration
Axiflow PSP60	CHL line shows weak coloration
Axiflow PSP60+	CHL line shows weak coloration
Axiflow PSP70	CHL line shows weak coloration
SR Bioera GF-BS-05	Fast flow, strong adhesive to backing card
MDI FR-2 (0.7)	Fast flow, strong adhesive to backing card
MDI FR-2 (0.8)	Fast flow, strong adhesive to backing card
MDI FR-1 (0.35)	Liquid did not reach CL
MDI FR-1 (0.6)	Liquid did not reach CL
Ahlstrom 601	Fast flow, but weak adhesive to backing card
Ahlstrom 142	CL shows weak coloration
Ahlstrom 121	Weak adhesive to backing card
Ahlstrom 222	Fast flow, strong adhesive to backing card
Ahlstrom 237	CL shows weak coloration
Ahlstrom 238	Fast flow, strong adhesive to backing card
Ahlstrom 270	Very high liquid absorbance, no liquid left in well
Ahlstrom 320	Very high liquid absorbance, no liquid left in well
Ahlstrom 440	CL shows weak coloration
Ahlstrom 319	CL shows weak coloration
Ahlstrom 1600	Fast flow, strong adhesive to backing card
Ahlstrom 1662	Fast flow, strong adhesive to backing card
Ahlstrom 1663	CL shows weak coloration
Ahlstrom 1667	CHL line shows weak coloration, liquid did not reach CL

**Table 2:** Summary of detergent effects on the TL and CL performance.

Application Site	Detergent	Notes
BSA-TET	0.2% CHAPS	Improved TL intensity (2–61%) and flow quality
BSA-click-AMP	0.2% Tween-80	
BSA-CHL	0.2% Tween-80	
BSA-click-STR	0.2% Tween-80	
GNP-conjugate	2% Triton X-100	Improved the CL intensity (+102%) in high-fat milk samples. Cannot be used simultaneously with TL detergent due to TL discoloration.

**Table 3:** Tested excipient formulations for lyophilization and the resulting overall desirability scores.

No.	Composition	D_j_
1	5% mannitol + 1% trehalose + 1% cyclodextrin	0.00
2	5% mannitol + 1% trehalose + 2.5% cyclodextrin	0.68
3	5% mannitol + 1% trehalose + 5% cyclodextrin	0.96
4	5% mannitol + 2.5% trehalose + 1% cyclodextrin	0.43
5	5% mannitol + 5% trehalose + 1% cyclodextrin	0.29
6	5% mannitol + 1% trehalose + 1% cyclodextrin + 1% sucrose	0.70
7	5% mannitol + 1% trehalose + 1% cyclodextrin + 2.5% sucrose	0.00
8	5% mannitol + 1% trehalose + 1% cyclodextrin + 5% sucrose	0.48
9	5% mannitol + 1% trehalose + 1% cyclodextrin + 1% dextran	0.70
10	5% mannitol + 1% trehalose + 1% cyclodextrin + 2.5% dextran	0.00
11	5% mannitol + 1% trehalose + 1% cyclodextrin + 5% dextran	0.46
12	1% trehalose + 1% cyclodextrin	0.30

**Table 4:** Sensitivity of the developed assay relative to the established MRLs (ng/mL).

Antibiotic	MRL TR CU	MRL China	MRL EU	vLOD	iLOD	LOQ
β-Lactams						
Ampicillin	4	4	4	4	1.9	3.5
Penicillin G	4	4	4	4	1.4	3.4
Cloxacillin	30	30	30	10	1.7	4.9
Amoxicillin	4	4	4	4	1.2	3.0
Ceftiofur	100	100	100	100	20.6	52.8
Aminoglycosides						
Streptomycin	200	200	200	50	0.5	3.0
Tetracyclines						
Doxycycline	10	0	0	10	0.7	4.4
Chlortetracycline	10	100	100	10	4.2	9.7
Oxytetracycline	10	100	100	1	0.3	0.9
Tetracycline	10	100	100	5	0.5	1.5
Amphenicols						
Chloramphenicol	0.3	0	0	0.3	0.2	0.3

## Data Availability

Data are contained within the article.

## Supplementary Materials

The following supporting information can be downloaded at: https://www.mdpi.com/article/10.3390/bios15090592/s1, Figure S1: ^1^H-NMR spectrum of **3** in CDCl_3_; Figure S2: ^1^H-NMR spectrum of **5** in CDCl_3_; Figure S3: ^1^H-NMR spectrum of **5** in DMSO−d_6_; Figure S4: MALDI-TOF MS of the BSA-TET, BSA-STR, BSA-CHL, and BSA-AMP conjugates; Figure S5: MALDI-TOF MS of the BSA-2IT and BSA-click-AMP conjugates; Figure S6: MALDI-TOF MS of the BSA-azide, BSA-click-CHL, and BSA-click-STR conjugates; Figure S7: (a) Spectrum of synthesized GNPs; (b) effect of GNPs size on the response in LFA. Values shown are the means of three runs (n=3); Figure S8: Synthesis scheme of CHL hapten **5**; Figure S9: Comparison of flow rate of test strips in low-fat milk samples depending on the presence of detergents in BSA–hapten conjugate application buffer; Figure S10: Testing the tolerance of leading sample pad materials for fat content in milk. Values shown are means of three runs (n=3); Figure S11: Effect of detergent presence on the intensity of the CL and TLs when analyzing milk with a high fat content. 1—Detergents were present only in the BSA–antibiotic application buffer. 2—Lyophilized gold conjugate contains 2% Triton X-100; detergents present in the protein–hapten application buffer. 3—Lyophilized gold conjugate contains 2% Triton X-100; Figure S12: Optimization of the conjugate concentration: (a) BSA-TET, (b) BSA-CHL, (c) BSA-click-STR, (d) BSA-click-AMP. Selected optimal concentrations are marked with a check mark. Values shown are the means of three runs (n=3); Figure S13: Concentration optimization of (a) anti-TET Ab, (b) anti-CHL Ab, (c) anti-STR Ab, and (d) PBP. Selected optimal concentrations are marked with a check mark. Values shown are the means of three runs (n=3); Figure S14: Cross-reactivity evaluation of test line signals in the presence of unrelated antibiotics. Values shown are the means of three runs (n=3).
