# Supplementary material for: Development of a Multiplex Lateral Flow Immunoassay for the Detection of Antibiotics in Milk Utilizing Lyophilized Gold Nanoparticle Conjugates
Source: Biosensors (Basel). 2025 Sep 9;15(9):592. doi: 10.3390/bios15090592 (PMC12467014; doi:10.3390/bios15090592)
Supplement: Supplementary file 1 [file biosensors-15-00592-s001.zip › biosensors-3795357-supplementary.pdf]

Supplementary Materials: Development of a Multiplex Lateral Flow Immunoassay for the Detection of Antibiotics in Milk Utilizing Lyophilized Gold Nanoparticle Conjugates

Ivan V. Maksin [1,2,*], Azhar Kuandykova [2], Darya I. Polyakova [2,3], Viktoriia A. Kesareva [2,3], Timofei A. Luzyanin [1], Vladislav S. Ivanov [2], Evgeniia I. Simonova [2,4], German A. Khunteev [2] and Yuliya G. Kirillova [1]

Synthesis of chloramphenicol hapten **5**

N-(tert-Butoxycarbonyl)prop-2-yn-1-yloxyethylamine **2**

In a three-necked round-bottom flask under an argon atmosphere, sodium hydride (0.12 g, 3.1 mmol, 1.0 equiv., 60% dispersion in mineral oil) was suspended in anhydrous THF (15 mL) and cooled to 0 °C using an ice bath. A solution of N-Boc-ethanolamine (0.50 g, 3.1 mmol, 1.0 equiv.) in THF (5 mL) was added dropwise via a dropping funnel. The reaction mixture was then allowed to warm to room temperature and stirred for 10 minutes. After cooling again to 0 °C, propargyl bromide (0.37 mL, 3.4 mmol, 1.1 equiv., 80% in toluene) in THF (2 mL) was added dropwise. The reaction mixture was gradually warmed to room temperature and stirred overnight. Upon completion, the solvent was removed under reduced pressure. The residue was dissolved in ethyl acetate (20 mL) and washed successively with distilled water (15 mL), saturated aqueous $NaHCO_3$ (15 mL), and saturated NaCl solution (10 mL). The organic layer was dried over anhydrous Na_2SO_4, filtered, and concentrated under reduced pressure. The crude product was purified by column chromatography using ethyl acetate/petroleum ether (1:4) as the eluent. Yield: 430 mg (70%). R_f = 0.40 (ethyl acetate/petroleum ether, 1:4).

^{1}H NMR (300 MHz, Chloroform-d) δ 4.90 (s, br, 1H, Boc-NH), 4.15 (d, J = 2.4 Hz, 2H, $O-CH_2-CCH$), 3.58 (t, J = 5.2 Hz, 2H, $NH-CH_2-CH_2$), 3.33 (q, J = 5.2 Hz, 2H, $NH-CH_2-CH_2$), 2.44 (t, J = 2.4 Hz, 1H, CCH), 1.44 (s, 9H, Boc).

Trifluoroacetate of propargyl-2-oxyethylamine **3**

A solution of Boc-protected amine **2** (0.15 g, 0.75 mmol, 1.0 equiv.) in dry CH_2Cl_2 (5 mL) was prepared in a single-neck round-bottom flask under an argon atmosphere. Triisopropylsilane (0.3 mL) was added to the solution, and the reaction mixture was cooled to 0 °C in an ice bath. TFA (5 mL) was then added dropwise. The mixture was allowed to warm to room temperature and stirred for 1 hour. After completion of the reaction, the solvents were removed under reduced pressure. The residue was co-evaporated with toluene and then with diethyl ether. The resulting viscous oil was used directly in the synthesis of hapten **5** without further purification.

Chl-succinate-PEG1-alkyne **5**

In a three-neck round-bottom flask under an argon atmosphere, the crude oil containing trifluoroacetate of propargyl-2-oxyethylamine **3** (0.16 g, 0.75 mmol, 2.1 equiv., 100% yield), sodium chloramphenicol succinate **4** (0.16 g, 0.36 mmol, 1.0 equiv.), 1-hydroxybenzotriazole (0.04 g, 0.18 mmol, 0.5 equiv.), and 4-dimethylaminopyridine (0.07 g, 0.54 mmol, 1.5 equiv.) was dissolved in DMF (5 mL). The reaction mixture was cooled to 0 °C in an ice bath, and triethylamine (0.11 mL, 0.75 mmol, 2.1 equiv.) was added dropwise. Subsequently, dry EDC (0.104 g, 0.54 mmol, 1.5 equiv.) were added. The mixture was allowed to warm to room temperature and stirred overnight. Upon completion, the solvent was removed under reduced pressure using a rotary evaporator connected to an

oil pump. The residue was dissolved in ethyl acetate (20 mL) and washed sequentially with distilled water (15 mL), 15% aqueous citric acid solution (15 mL), saturated aqueous $NaHCO_3$ solution (15 mL), and saturated NaCl solution (10 mL). The organic layer was dried over anhydrous Na_2SO_4, filtered, and concentrated under reduced pressure. The crude product was purified by column chromatography using ethyl acetate as the eluent. Yield: 113 mg (62%). R_f= 0.70 (CH_2Cl_2/MeOH, 5:0.2), 0.30 (ethyl acetate).

^{1}H NMR (300 MHz, Chloroform-d) δ 8.19 (dq, J = 9.0, 2.5 Hz, 2H, Ph−NO_2), 7.64–7.55 (m, 2H, Ph−NO_2), 7.24 + 7.20 (d, J = 4.3 Hz, 1H + s, 1H, NH−CH_2 + NH-CH), 5.98 (s, br, 1H, $CHCl_2$), 5.82 (d, J = 4.6 Hz, 1H, CH-OH), 5.20 (d, br, J = 4.8 Hz, 1H, OH), 4.51 (dd, J = 10.9, 6.2 Hz, 1H, CH_2−O−succ), 4.37 (dtd, J = 8.6, 5.3, 2.5 Hz, 1H, NH-CH), 4.29 (dd, J = 10.8, 4.8 Hz, 1H, CH_2−O−succ), 4.15 (d, J = 2.5 Hz, 2H, O−CH_2−CCH), 3.59 (q, J = 4.9 Hz, 2H, CO−CH_2−CH_2−CO), 3.46 (q, J = 5.2 Hz, 2H, CO−CH_2−CH_2−CO), 2.70–2.55 (m, 4H, NH−CH_2−CH_2), 2.46 (t, J = 2. Hz, 1H, CCH).

^{1}H NMR (300 MHz, DMSO-d_6) δ 8.41 (d, br, J = 8.8 Hz, 1H, NH-CH), 8.20–8.12 (m, 2H, Ph-NO_2), 7.89 (t, J = 5.3 Hz, 1H, NH-CH_2), 7.64 (d, J = 8.7 Hz, 2H, Ph-NO_2), 6.44 (d, J = 0.7 Hz, 1H, $CHCl_2$), 6.13 (d, J = 4.6 Hz, 1H, CH-OH), 5.05–5.00 (m, 1H, OH), 4.29–4.16 + 4.16–4.04 (m, 3H + m, 2H, NH-CH + CH_2-O-succ + O-CH_2-CCH), 3.45 (t, J = 5.8 Hz, 2H, CO−CH_2−CH_2−CO), 3.36 (q, J = 3.0, 2.4 Hz, 1H, CCH), 3.19 (s, 2H, CO−CH_2−CH_2−CO), 2.46 + 2.37 (s, 2H + t, J = 7.2 Hz, 2H, NH−CH_2−CH_2).

^{13}C NMR (75 MHz, DMSO-d_6) δ 172.14, 170.77, 163.60, 150.21, 146.61, 127.44, 122.86, 80.21, 77.11, 69.46, 67.93, 66.26, 62.93, 57.34, 53.49, 38.33, 29.65, 28.93.

*Synthesis of streptomycin hapten **6***

To 0.85 mL of a 0.45 M Na_2CO_3 solution, streptomycin sulfate (408 mg, 0.56 mmol) and propargyloxypropionic acid hydrazide (30 mg, 0.21 mmol) were added, and the mixture was incubated for 24 hours at 4 °C. The reaction progress was monitored by TLC using a dichloromethane/methanol (40:1) system by the disappearance of the starting material (R_f = 0.3). After elution, the plates were treated with ninhydrin solution and heated for visualization. The concentration of the resulting streptomycin hapten **6** in solution was estimated to be 0.247 M.

Synthesis of BSA-CHL

To 4.2 mL of 10 mM MES buffer (pH 6.0), 17 mg of the sodium succinate salt of chloramphenicol, 15 mg of EDC, and 10 mg of NHS were added and stirred for 1 hour at room temperature. Then, 1 mL of BSA solution (50 mg/mL) in 10 mM MES buffer (pH 6.0) was added to the reaction mixture, followed by stirring overnight. The conjugate was purified by dialysis against PBS buffer. The resulting conjugate was labeled as BSA-CHL.

Synthesis of BSA-click-CHL

To a solution of 0.5 mL BSA-azide at a concentration of 8.46 mg/mL (0.063 μmol, 1 equiv.), 0.1 mL of 1 M HEPES buffer (pH 7.2) and 13 μL of chloramphenicol hapten **5** in DMF (0.63 μmol, 10 equiv.) were added. In a separate tube, 6.25 μL of 20 mM $CuSO_4$ solution (0.125 μmol, 2 equiv.) and 12.5 μL of 50 mM THPTA solution (0.625 μmol, 10 equiv.) were mixed. The entire volume of the resulting complex was then transferred to the BSA-azide/hapten mixture. Subsequently, 50 μL of 100 mM hydroxylamine hydrochloride (5 μmol, 80 equiv.) was added. The reaction mixture was flushed with argon and stirred for 24 hours. The conjugate was purified by dialysis against PBS buffer. The resulting conjugate was labeled as BSA-click-CHL.

Synthesis of BSA-AMP

In 4.2 mL of 10 mM MES buffer (pH 6.0), 10 mg of sodium ampicillin, 8 mg of EDC, and 10 mg of NHS were mixed. The reaction mixture was incubated for 1 hour at room temperature on an orbital shaker. Subsequently, 0.25 mL of BSA solution (10 mg/mL) was added, and the mixture was incubated for 24 hours at 4 °C under continuous stirring. The conjugate was purified by dialysis against PBS buffer. The resulting conjugate was labeled as BSA-AMP.

BSA-click-AMP

To a solution of 0.1 M Na_2CO_3 (pH 9), 43 mg of BSA and 1.7 mg of 2-iminothiolane were added to give the BSA-2IT conjugate. Separately, ampicillin sodium salt was dissolved in 0.1 M Na_2CO_3 (pH 9), and 11 mg of Sulfo-SMCC was added. Both solutions were incubated for 1 hour and then mixed together, followed by the addition of 0.6 mL of 0.5 M NaH_2PO_4, and incubated overnight. The conjugate was purified by dialysis against PBS buffer. The conjugate was purified by dialysis against PBS buffer. The resulting conjugate was labeled as BSA-click-AMP.

BSA-GA-STR

In a glass beaker placed on a platform shaker, 118 mg of streptomycin sulfate and 45 mg of BSA were dissolved in 10 mL of 100 mM carbonate–bicarbonate buffer (pH 9.5). Then, 0.5 mL of a 0.1% aqueous solution of glutaraldehyde was added dropwise under constant stirring. The reaction mixture was stirred at room temperature for 5 hours. A faint yellow-green coloration of the initially colorless solution was observed.

After incubation, 50 μL of a freshly prepared 10% sodium borohydride solution was added. Within a few minutes, the evolution of gas bubbles and foam formation was observed. After the gas release ceased and the solution became colorless, the resulting conjugate was purified by dialysis against PBS buffer. The resulting conjugate was labeled as BSA-GA-STR.

BSA-click-STR

To a solution of 0.5 mL BSA-azide at a concentration of 8.46 mg/mL (0.063 μmol, 1 equiv.), 0.1 mL of 1 M HEPES buffer (pH 7.2) and 13 μL of streptomycin hapten **6** (3.211 μmol, 50 equiv.) were added. In a separate tube, 6.25 μL of 20 mM $CuSO_4$ solution (0.125 μmol, 2 equiv.) and 12.5 μL of 50 mM THPTA solution (0.625 μmol, 10 equiv.) were mixed. The entire volume of the resulting complex was then transferred to the BSA-azide/STR-alkyne mixture. Subsequently, 50 μL of 100 mM hydroxylamine hydrochloride (5 μmol, 80 equiv.) was added. The reaction mixture was flushed with argon and stirred for 24 hours. The conjugate was purified by dialysis against PBS buffer. The resulting conjugate was labeled as BSA-click-STR.

BSA-TET

A mixture of 8 mL of BSA solution (18.75 mg/mL), 2 mL of 3 M sodium acetate, and 12 mL of tetracycline hydrochloride solution (8.3 mg/mL in water) was prepared. While stirring, 3.75 mL of 37% formaldehyde solution was added dropwise. The reaction vessel was protected from light with aluminum foil, and the mixture was stirred at room temperature for 6 hours. The conjugate was purified by dialysis against PBS buffer. The resulting conjugate was labeled as BSA-TET.

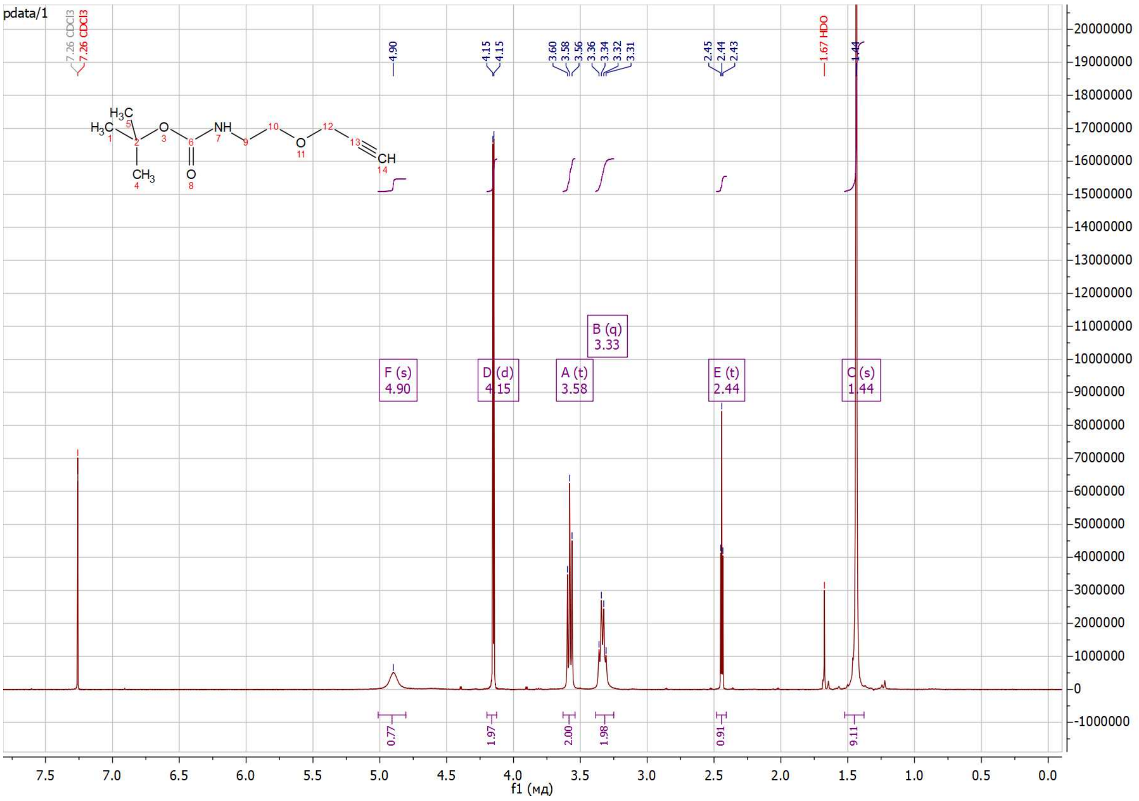

Figure S1. ^{1}H-NMR spectrum of **3** in $CDCl_3$.

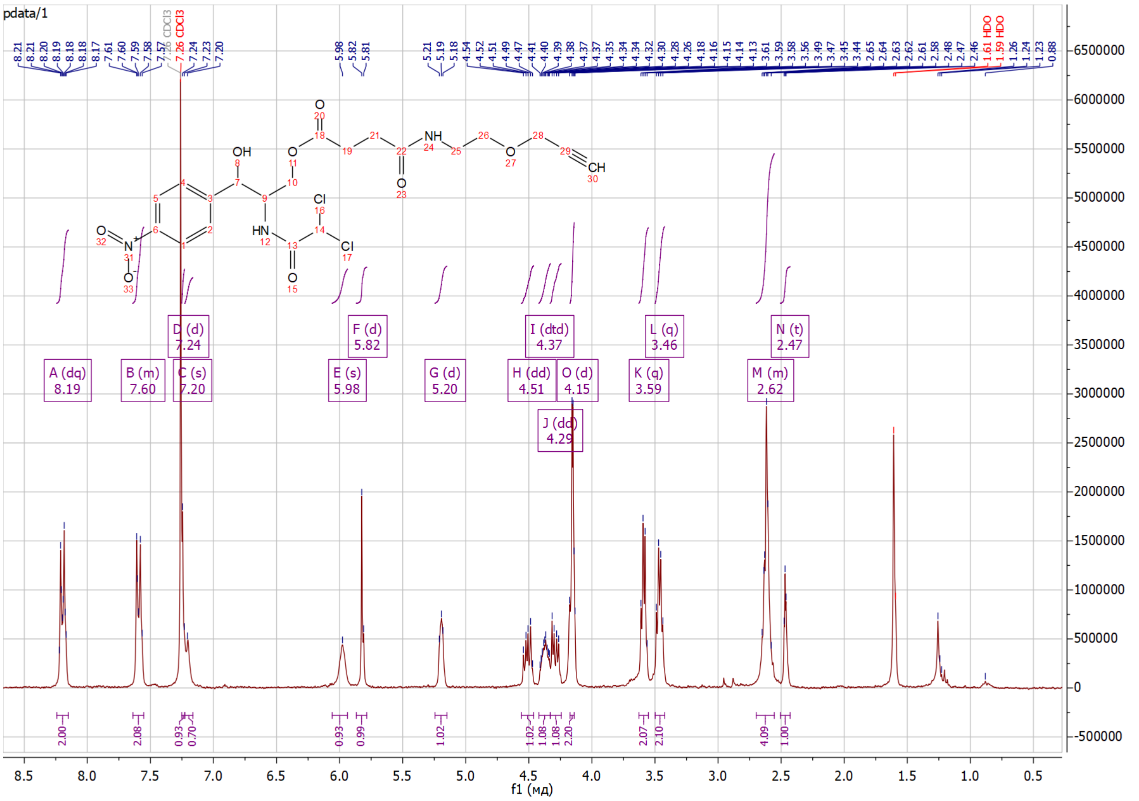

Figure S2. ^{1}H-NMR spectrum of **5** in $CDCl_3$.

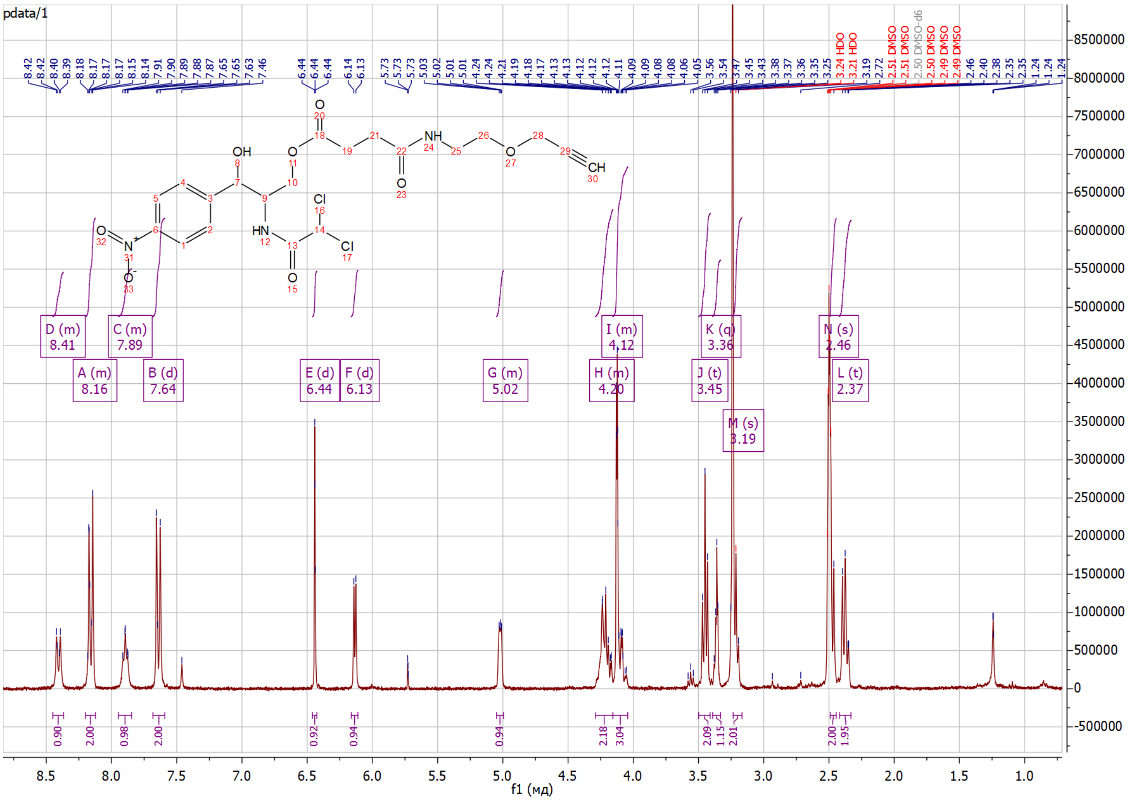

Figure S3. ^{1}H-NMR spectrum of **5** in DMSO−d_6.

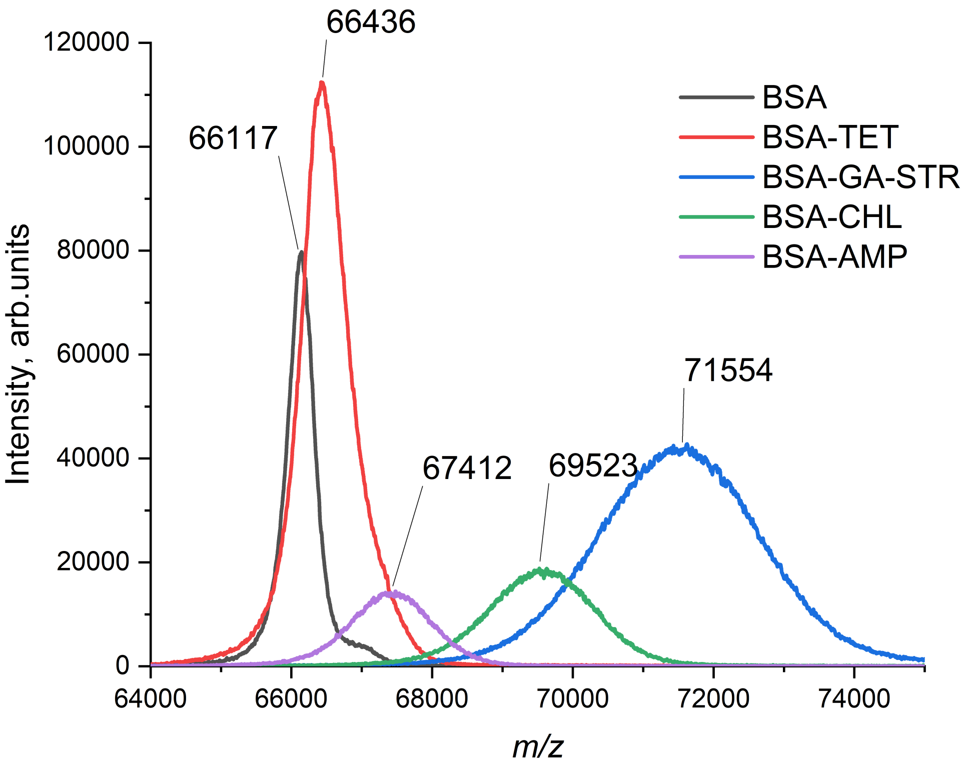

Figure S4. MALDI-TOF MS of the BSA-TET, BSA-STR, BSA-CHL, and BSA-AMP conjugates.

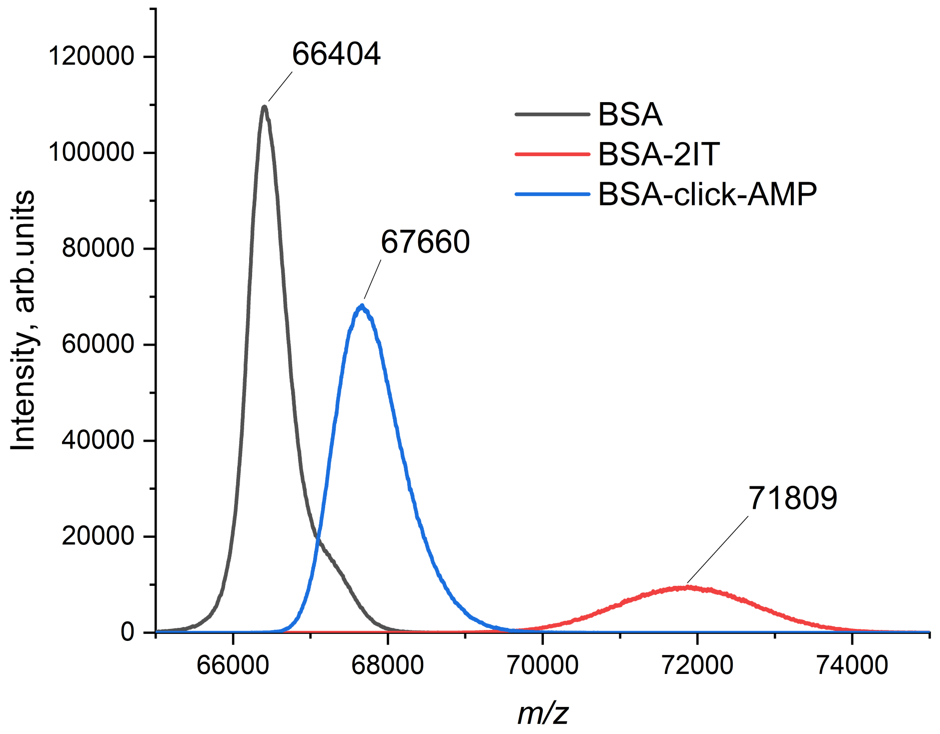

Figure S5. MALDI-TOF MS of the BSA-2IT and BSA-click-AMP conjugates.

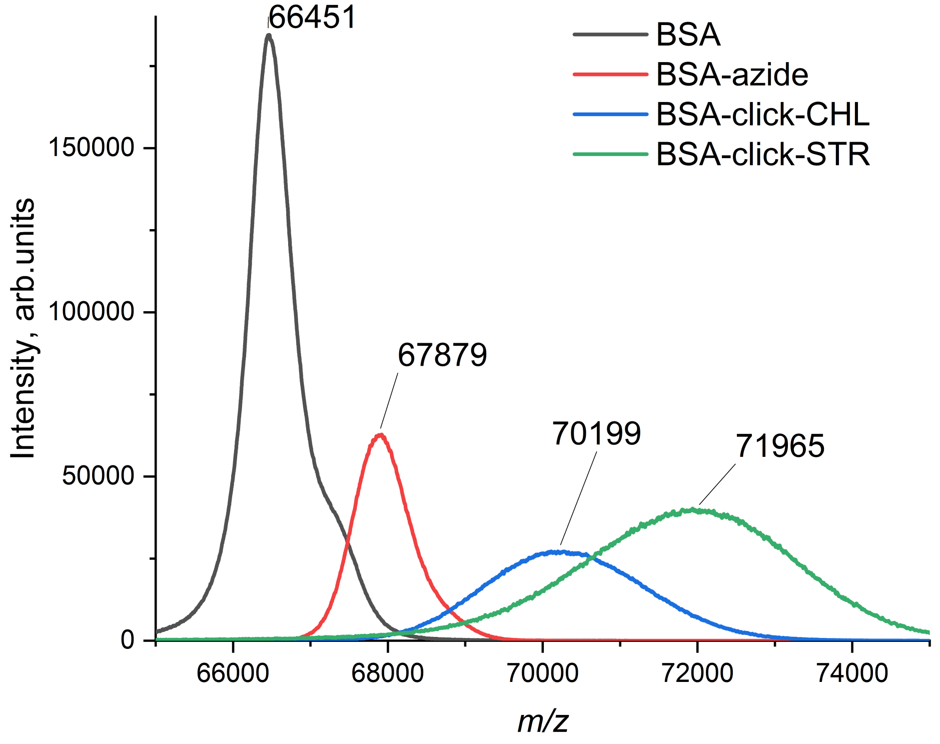

Figure S6. MALDI-TOF MS of the BSA-azide, BSA-click-CHL, and BSA-click-STR conjugates.

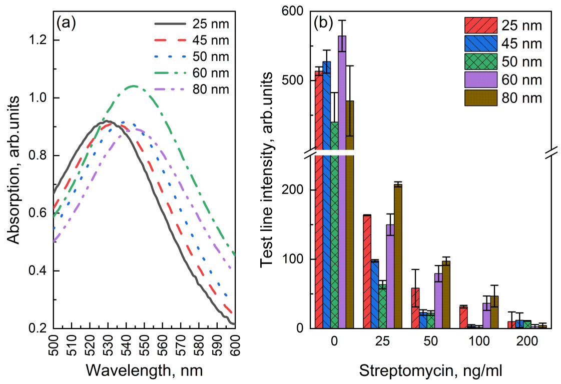

Figure S7. (a) Spectrum of synthesized GNPs; (b) effect of GNPs size on the response in LFA. Values shown are the means of three runs ($n = 3$).

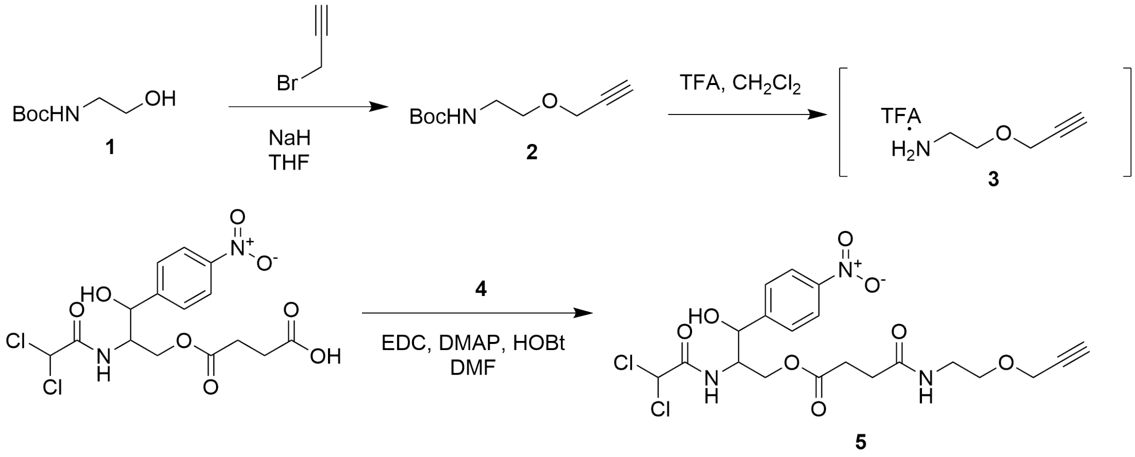

Figure S8. Synthesis scheme of CHL hapten **5**.

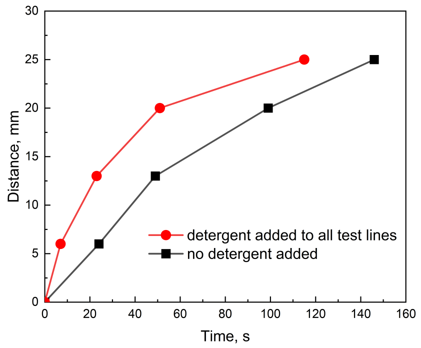

Figure S9. Comparison of flow rate of test strips in low-fat milk samples depending on the presence of detergents in BSA–hapten conjugate application buffer.

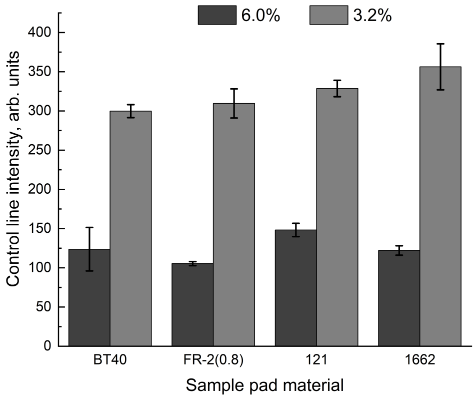

Figure S10. Testing the tolerance of leading sample pad materials for fat content in milk. Values shown are means of three runs ($n = 3$).

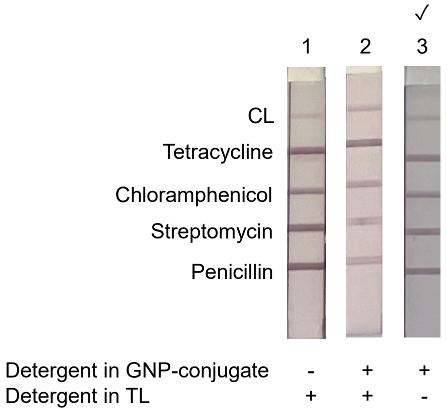

Figure S11. Effect of detergent presence on the intensity of the CL and TLs when analyzing milk with a high fat content. 1—Detergents were present only in the BSA–antibiotic application buffer. 2—Lyophilized gold conjugate contains 2% Triton X-100; detergents present in the protein–hapten application buffer. 3—Lyophilized gold conjugate contains 2% Triton X-100.

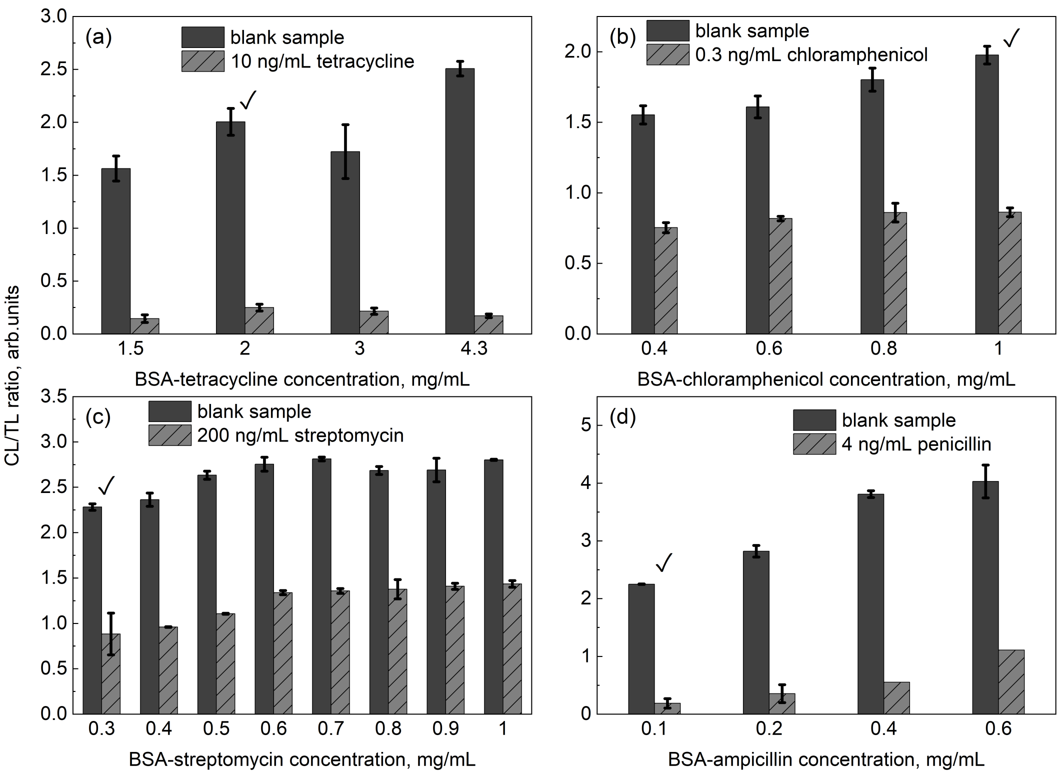

Figure S12. Optimization of the conjugate concentration: (a) BSA-TET, (b) BSA-CHL, (c) BSA-click-STR, (d) BSA-click-AMP. Selected optimal concentrations are marked with a check mark. Values shown are the means of three runs ($n = 3$).

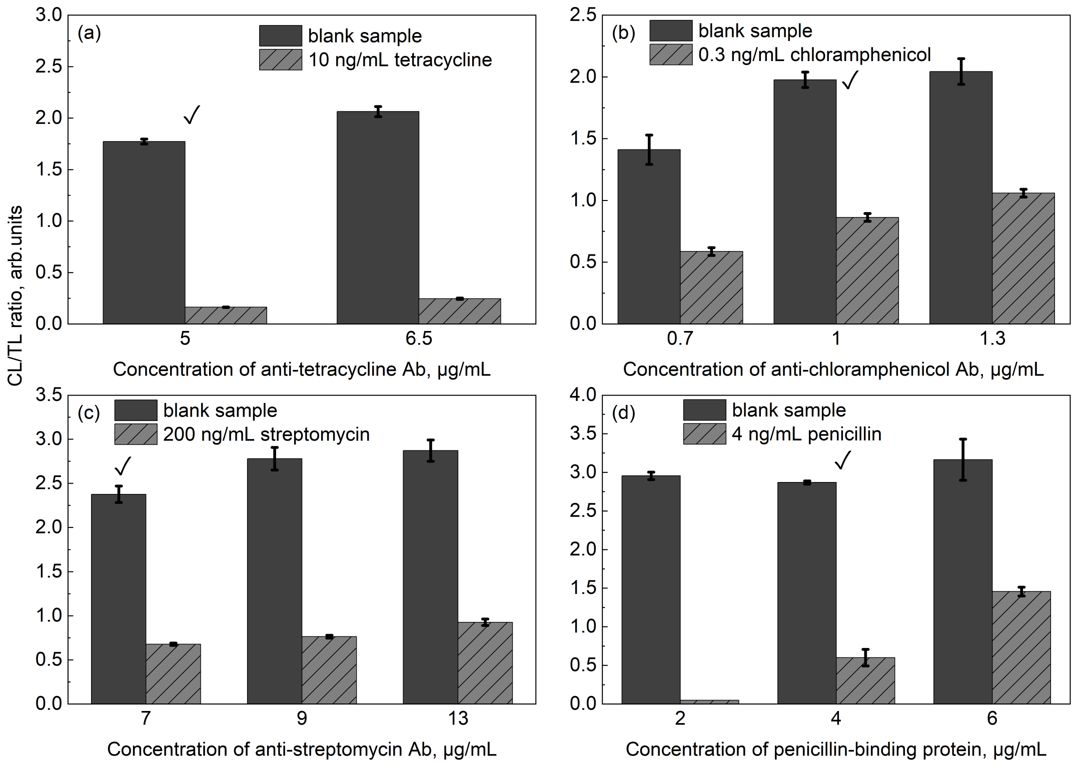

Figure S13. Concentration optimization of (a) anti-TET Ab, (b) anti-CHL Ab, (c) anti-STR Ab, and (d) PBP. Selected optimal concentrations are marked with a check mark. Values shown are the means of three runs ($n = 3$).

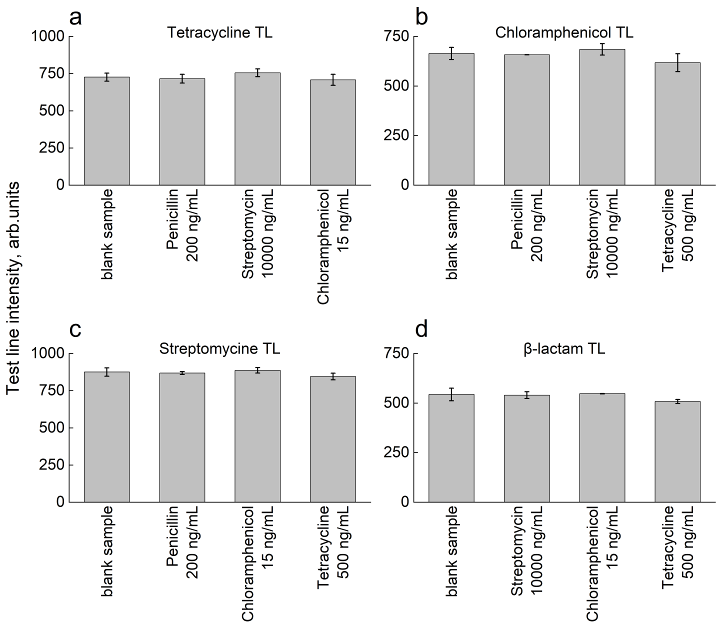

Figure S14. Cross-reactivity evaluation of test line signals in the presence of unrelated antibiotics. Values shown are the means of three runs ($n = 3$).
